# *Lactobacillus plantarum* MYL26 induces endotoxin tolerance phenotype in Caco-2 cells

**DOI:** 10.1186/1471-2180-13-190

**Published:** 2013-08-10

**Authors:** Yi-Heng Chiu, Ying-Chen Lu, Chu-Chyn Ou, Shiao-Lin Lin, Chin-Chi Tsai, Chien-Tsai Huang, Meei-Yn Lin

**Affiliations:** 1 Department of Food Science and Biotechnology, National Chung Hsing University, 250 Kuokuang Road, Taichung 40227, Taiwan; 2 Department of Food Science, National Chiayi University, Chiayi City, Taiwan; 3 School of Nutrition, Chung Shan Medical University, Taichung, Taiwan; 4 Department of Nutrition, Chung Shan Medical University Hospital, Taichung, Taiwan; 5 Department of Neurology, Chong Guang Hospital, MiaoLi County, Taiwan

**Keywords:** *Lactobacillus plantarum* MYL26, Lipopolysaccharide, Caco-2 cells

## Abstract

**Background:**

Crohn's disease and ulcerative colitis are the major types of chronic inflammatory bowel disease occurring in the colon and small intestine. A growing body of research has proposed that probiotics are able to attenuate the inflammatory symptoms of these diseases *in vitro* and *in vivo*. However, the mechanism of probiotic actions remains unclear.

**Results:**

Our results suggested *Lactobacillus plantarum* MYL26 inhibited inflammation in Caco-2 cells through regulation of gene expressions of *TOLLIP*, *SOCS1*, *SOCS3*, and *IκBα*, rather than *SHIP-1* and *IRAK-3*.

**Conclusions:**

We proposed that live/ heat-killed *Lactobacillus plantarum* MYL26 and bacterial cell wall extract treatments impaired TLR4-NFκb signal transduction through *Tollip*, *SOCS-1* and *SOCS-3* activation, thus inducing LPS tolerance. Our findings suggest that either heat-killed probiotics or probiotic cell wall extracts are able to attenuate inflammation through pathways similar to that of live bacteria.

## Background

Inflammatory bowel disease (IBD) comprises a collection of disorders, which mainly include Crohn's disease and ulcerative colitis. These disorders cause abdominal pain, vomiting, diarrhea, and gastrointestinal (GI) inflammation [1]. To date, no effective therapy has been developed and patients may have a reduced quality of life even under proper management. It has been shown that factors related to IBD include acquired factors (e.g., smoking and diet), pathogens, genetic factors, and irregular immune system [2]. Over the past decades, the homeostatic functions of microflora on host GI tract have attracted much attention because growing numbers of clinical studies have suggested that probiotics exhibit anti-inflammatory effects on IBD patients [3,4]. Arseneau et al. [5] suggested that innate immune responses play an equally significant, even more primary character compared with adaptive immune responses in IBD initiation and progression due to the observation that probiotics elicit anti- inflammatory effects in the GI tract by means of mucosal innate immune system stimulation, instead of suppression. In this study, we put efforts on addressing the interactions between probiotics and intestinal epithelial cells, the mechanism different from the conventionally dichotomous Th1/Th2 cytokine paradigm.

Probiotics have no pharmacological actions confirmed, but numerous benefits have been proposed, such as immunomodulation [6,7], antioxidant capacities [8], hepatoprotective effects [9], maintenance of commensal microflora [10], pathogen antagonization [11], anti-allergic effects [12,13] and decreased endotoxin level in plasma [14]. *Lactobacillus plantarum*, one of the most commonly used probiotics, is a member of the aerotolerant group of lactobacilli found in several fermented foods [15]. It is also one of the dominant *Lactobacillus* species in the hosts’ intestinal tract. Recent studies have shown that some strains of *Lactobacillus plantarum* attenuate inflammation induced by *Shigella flexneri* peptidoglycan by inhibiting nuclear factor kappa-light-chain-enhancer of activated B cells (NFκB), inactivating mitogen-activated protein kinase (MAPK), and reducing NOD2 mRNA expression as well as protein levels, the actions which in turn lead to a decrease in pro-inflammatory cytokine secretion [16]. Moreover, van Baarlen et al. [17,18] demonstrated that even dead *L. plantarum* can exert beneficial functions protecting the host against the enormous array of commensal bacteria in the gut via epithelial crosstalk of mucosal interface microbiota. Their research team further investigated *in vivo* transcriptome responses to probiotics, the work shaping that different probiotic strains induced differential gene-regulatory networks and pathways in the human mucosa [19]. This provides advanced concept that not only live probiotics can exert beneficial effects, but also dead probiotics are able to modulate GI homeostasis. Second, because of strain-dependent properties, the anti-inflammation mechanism of single strains could not be extrapolated from other specific consequences without empirical evidence.

Systemic exposure to endotoxins accompanied with elevation of interleukin (IL)-6, IL-8 and IL-12 has been recognized as representative features of IBD progression [20,21]. Endotoxins are a family of molecules that bind to many pattern recognition receptors. One of the most dominant endotoxins is lipopolysaccharide (LPS). Previous exposure to LPS leads to cells hyporesponsive to subsequent challenge with LPS. This phenomenon is regarded as LPS tolerance.

LPS tolerance is typically associated with poor signal transduction in TLR4-NFκB pathway. TLR4 recognizes LPS from Gram-negative bacteria. Myeloid differentiation primary response gene 88 (Myd88) acts as a universal adapter protein used by TLRs (except for TLR3). Interleukin-1 receptor-associated kinase 1 (IRAK1) belongs to the serine/threonine protein kinase family. This kinase mediates the TLR4 downstream signaling transduction induced by LPS, forming a kinase complex required for the activation of NFκB. TNF receptor associated factor 6 (TRAF6) kinase mediates signal transduction from IRAK1, providing a link between IRAK1 and Mitogen-activated protein kinase 7 (TAK1). TAK1 activate IκB kinase (IKK), and thus plays a role in relaying signals to NFκB. IKK is an enzyme complex involved in IκBα phosphorylation, the action that gives rise to NFκB nuclear translocation [22].

It is well-studied that poor signal transduction in TLR4-NFκB pathway is mainly attributed to negative regulators [23]. Suppressors of cytokine signaling 1 (SOCS1) and suppressors of cytokine signaling 3 (SOCS3) are able to reduce JAK/STAT signal transduction, involving negative feedback to cytokine signaling [24]. Toll interacting protein (TOLLIP) interacts with many types of TLR signaling downstream pathways and potently inhibits the activity of IRAK after TLR activation. Overexpression of TOLLIP has been reported to inhibit inflammation in response to TLR4 signaling [25]. IL-1R-associated kinase 3 (IRAK3) suppresses the dissociation of IRAK1/4 from Myd88 and the connections among TRAF 6 complexes [26]. Phosphatidylinositol-3,4,5-trisphosphate 5-phosphatase 1 (SHIP1) hydrolyses phosphatidylinositol 3-kinase, hence interfering with TLR4-MyD88 signaling pathway [27].

Since attenuation of pro-inflammatory cytokines secretions is IBD therapeutic targets, In this study, we co-cultured human epithelial colorectal adenocarcinoma (Caco-2) cells with probiotics and then administered LPS, which induced TNF-α, IL-6, IL-8 and IL-12 secretion, to biologically mimic the inflammatory situation of IBD. With the purpose of determining how *L. plantarum* weakens the downstream signal transduction of TLR4, the mRNAs that encode proteins participating in TLR4-NF-κB pathway were detected by RT-qPCR. Five negative regulator genes, *SOCS1*, *SOCS3*, *TOLLIP*, *IRAK3* and *SHIP1,* which may result in inactivation of TLR4-NF-κB pathway, were also examined whether or not to be affected by probiotic treatment. Moreover, in order to explore which cellular parts contribute mostly to the anti-inflammatory properties, we tested the anti-inflammatory efficacies of live bacteria, heat-killed bacteria, cell wall extract, intracellular extract and bacterial genomic DNA in terms of negative regulator activation capacity.

## Methods

### Lactic acid bacterial strains

Isolation and identification of *Lactobacillus plantarum* from newborn infant feces and breast milk were performed in the Microbiology Laboratory of the Department of Food Science and Biotechnology of National Chung Hsing University, Taichung, Taiwan. Our preliminary data showed *L. plantarum* MYL26, *L. plantarum* MYL31, and *L. plantarum* MYL68 have better anti-inflammation abilities than those of other strains isolated in our laboratory.

### Ethics statement

The samples from infants and adult subjects were approved employing in this study by Jeng-Yuan Hsu, Chairman of Institutional Review Board of the Taichung Veterans General Hospital. We obtained informed consent from both adult subjects and these infants’ guardians for collection of sample.

### Preparation of cell wall, intracellular extracts and heat-killed lactic acid bacteria

All bacterial strains used in this study were stored at -80°C. *Lactobacillus plantarum* MYL26, *Lactobacillus plantarum* MYL31, and *Lactobacillus plantarum* MYL68 were cultured in MRS broth at 37°C for 16 h and collected by centrifugation at 2500 *g* for 8 min. For preparation of cell wall and intracellular extracts, cells were adjusted to 10^7^ cfu/mL, washed twice with deionized water and suspended in phosphate-buffered saline (PBS). FRENCH® Pressure Cells Press (Thermo Electron, Waltham, USA) was used for cell disruption. Cell wall was removed by centrifugation at 5000 *g* for 10 min, and the supernatant was filtered through 0.22 *μ*m filters as intracellular extract. The protein contents of intracellular extracts were adjusted to 1 mg/mL. The weight of cell wall extracts processed according to this protocol is about 10 ± 0.2 mg/10^7^ cfu. For preparation of heat-killed cells, cells were suspended in PBS and adjusted to 10^7^ cfu/mL followed by killing at 65°C for 30 min.

### Preparation of bacterial genomic DNA

Lactic acid bacteria genomic DNA was extracted by tissue and cell genomic DNA purification system (GeneMark, Taichung, Taiwan). Nucleic acid concentration was measured at a wavelength of 260 nm and adjusted to 10 μg/mL.

### Cell culture

Human intestinal epithelial-like cells (Caco-2) were obtained from the Bioresource Collection and Research Center (BCRC, Hsinchu, Taiwan) and cultured in Dulbecco’s modified Eagle’s medium (DMEM) supplemented with 10% heat-inactivated fetal bovine serum (FBS), penicillin (100 units/mL) and streptomycin (100 mg/mL) at 37°C in a humidified (95%) atmosphere with 5% CO_2_.

### Cytokine secretions by stimulation of Caco-2 cells with *L. plantarum* MYL26 followed by LPS challenge

Caco-2 cells (10^6^ cells/mL) were treated with live *L. plantarum* MYL26 (10^7^ cfu/mL), heat-killed bacteria (10^7^ cfu/mL), intracellular extracts (100 μg/mL), cell wall extracts (10 ± 0.2 mg/mL) and genomic DNA (1 μg/mL) at 37°C for 10 hours. After stimulation, cells were challenged with 1 μg/mL LPS for 18 hours. The supernatants were removed and IL-6, IL-8, IL-12p70 and TNF-α secretions were assayed by enzyme-linked immunosorbent assay (eBioscience ELISA system, California, USA).

### siRNA silencing technique

Silencing of human *SOCS1*, *SOCS3* and *TOLLIP* expressions was carried out in Caco-2 cells by using Dharmacon Human siGENOME® SMARTpool® siRNA Libraries for antisense oligonucleotides (AO) design. AO were transfected with DharmaFECT 2 reagent (Thermo Fisher Scientific, Massachusetts, USA) according to the manufacturer’s instructions. The siRNA experiment was conducted for 48 h and cells were collected to analyze total RNA for knockdown effect.

### RT-qPCR

RNA isolation was conducted using EZ-RNA total RNA isolation kit (Biological Industries, Beit Haemek, Israel). Reverse transcription was carried out according to manufacturer’s instruction (Bio-Rad iScript™ cDNA synthesis kit, USA). Comparisons of gene expressions via qPCR were performed by adopting the following primer designs: *SOCS3* (5′-CAA ATG TTG CTT CCC CCT TA-3′ and 5′-ATC CTG GTG ACA TGC TCC TC-3′), *SHIP1* (5′-TCC AGC AGT CTT CCT CAC CT-3′ and 5′-GCT TGG ACA CCA TGT TGA TG-3′), *IRAK3* (5′-GGG TGC CTG TAG CAG AGA AG-3′ and 5′-ATC TGG AGG AGC CAG GAT TT-3′), *SOCS1* (5′-CTG GGA TGC CGT GTT ATT TT-3′ and 5′-TAG GAG GTG CGA GTT CAG GT-3′), *TOLLIP* (5′-CCA CAG TGT GAG GGA TTG TG-3′ and 5′-TCT CCT TCT CAT GCC GTT CT-3′), *MyD88* (5′-GCA CAT GGG CAC ATA CAG AC-3′ and 5′-GAC ATG GTT AGG CTC CCT CA-3′), *IKKβ* (5′-GCT GCA ACT GAT GCT GAT GT-3′ and 5′- TGT CAC AGG GTA GGT GTG GA-3′), *TAK1* (5′-TTT GCT GGT CCT TTT CAT CC-3′ and 5′-TGC CCA AAC TCC AAA GA ATC-3′), *TLR4* (5′-TGA GCA GTC GTG CTG GTA TC-3′ and 5′-CAG GGC TTT TCT GAG TCG TC-3′), *IκBα* (5′-GCA AAA TCC TGA CCA GGT GT-3′ and 5′-GCT CGT CCT CTG TGA ACT CC-3′), *GAPDH* (5′-GAG TCA ACG GAT TTG GTC GT-3′ and 5′-TTG ATT TTG GAG GGA TCT CG-3′), *TRAF6* (5′-CTG CAA AGC CTG CAT CAT AA-3′ and 5′-GGG GAC AAT CCA TAA GAG CA-3′), *IRAK1* (5′-GGG TCC AGG TGC TTC TTG TA-3′ and 5′-TGC TAG AGA CCT TGG CTG GT-3′). Quantitative PCR was carried out according to the manufacturer’s protocol. After reverse transcription of mRNA, 5 μl of the reverse transcription product were added to a BioRad iCyclerTM PCR system containing 0.3 μM of each primer. One-fold QuantiTect SYBR Green PCR Master Mix was used as a fluorescent reporter (QuantiTect SYBR Green PCR, Qiagen). The condition was programmed as follows: (1) Denaturation at 94°C for 10 min; (2) Amplification for 40 cycles of denaturation at 94°C for 15 s, annealing at 55°C for 30 s, and extension at 72°C for 20 s.

### Cell viability assay

3-[4,5-dimethyl-2-thiazolyl]-2,5-diphenyl-2H-tetrazolium bromide (MTT) assay, which is based on the cleavage of the tetrazolium salt by mitochondrial dehydrogenases in viable cells. In order to determine toxicity concentration, approximately 10^5^ cells were plated onto each well of 96-well plates for 24 h, followed by treatment with different probiotic agents for 6, 8, 10, 12 and 14 hours. After incubation, 200 mL of MTT solution (0.5 mg/mL) were added to each well for 4 h after washing by PBS. Finally, the supernatant was removed and 200 μL of dimethyl sulphoxide (DMSO) were added to each well to dissolve the dark blue formazan crystals. The absorbance was measured by ELISA plate reader (Jupiter, ASYS Hitech, Austria) at 570 nm. To compare the results, the relative cell viability was expressed as the mean percentage of viable cells compared with untreated cells (100%).

### Statistical analysis

Each value is the mean of triplicate experiments in each group. Means comparison was carried out by Student's t-test. *P* < 0.05 was considered significantly different.

## Results

### *Lactobacillus plantarum* MYL26/ MYL31/ MYL68 treatment did not affect the Caco-2 cell viability within 10 hours

**Figure 1:**
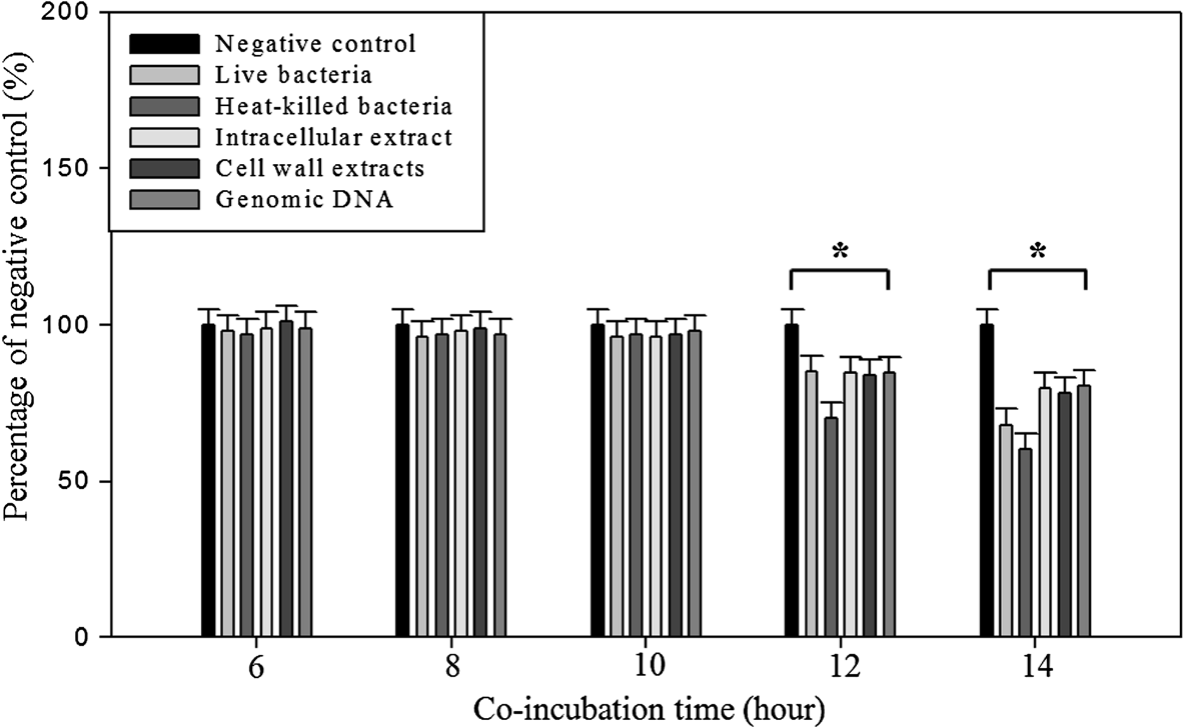
**Approximately 1 × 10**^**5 **^**cells were plated onto 96-well plates for 24 h, followed by treatment with live/ heat-killed *L. plantarum *MYL26 (*L. plantarum *MYL31/ MYL68 data not shown) and different cellular parts for 6, 8, 10, 12 and 14 hours.** Symbol * represents P-value smaller than 0.05 analyzed by *t*-test in comparison with negative control group. (n = 3). Negative control: Caco-2 cells were not treated with probiotics.

Due to excellent lactic acid production capacities of *Lactobacillus plantarum*, we perform MTT assay to assess the most appropriate incubation time. As Figure 1 showed, cell viability was not influenced within 10 hours. Incubated with 12 and 14 hours, Caco-2 cell viability showed significant decrease. As a result, we co-cultured Caco-2 cells and *Lactobacillus plantarum* for 10 hours in the following experiments.

### *Lactobacillus plantarum* attenuates LPS-induced cytokine secretion

**Figure 2:**
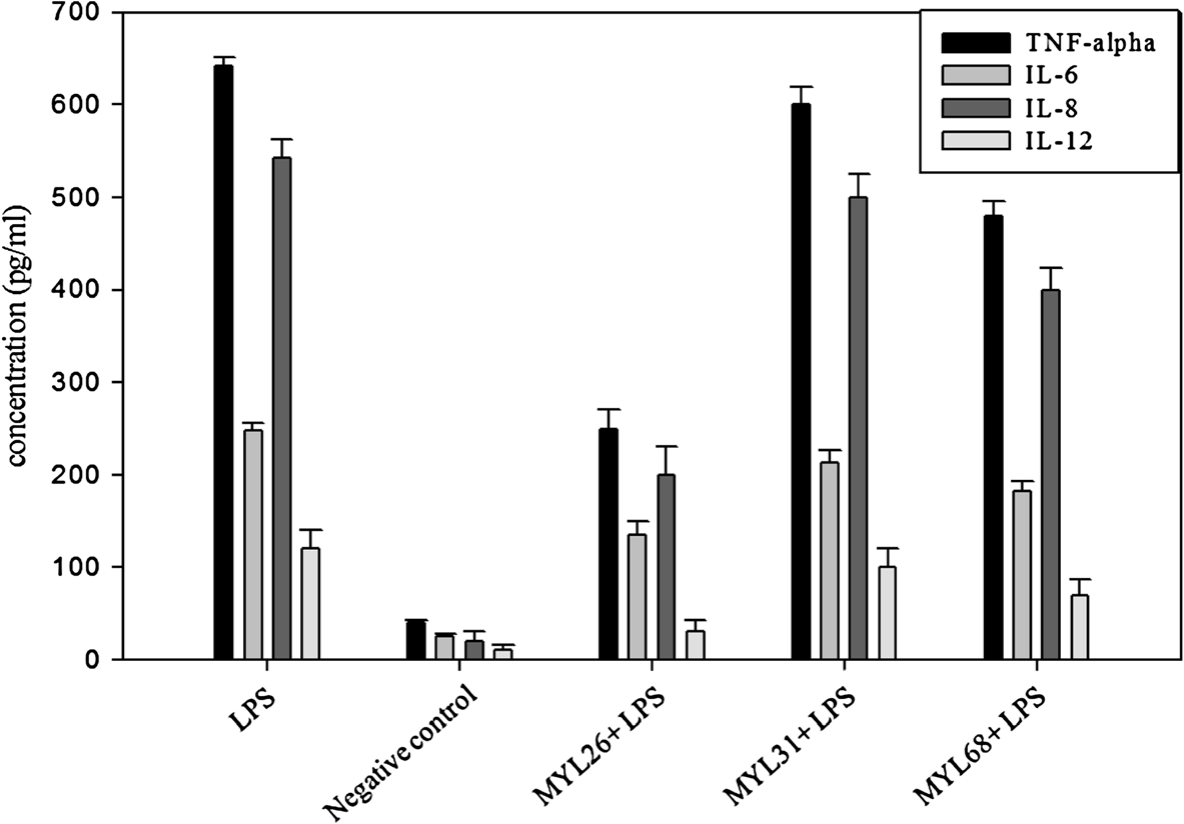
**Caco-2 cells (10**^**6 **^**cells/mL) were treated with live *L. plantarum *MYL26/ MYL31/ MYL68 (10**^**7**^ **cfu/mL) at 37°C for 10 hours, followed by 1 μg/mL LPS challenge.** Negative control: Caco-2 cells were not treated with LPS and probiotics. (Cytokine secretion baseline).

Three different strains of *Lactobacillus plantarum* (MYL26, MYL31 and MYL68) were tested and the most potent strain, in terms of refractoriness to subsequent LPS stimulation, was selected. As shown in Figure 2, *L. plantarum* MYL26 attenuated TNF-α, IL-6, IL-8, and IL-12 production more effectively than those of other strains.

### *Lactobacillus plantarum* MYL26 attenuates downstream signal transduction of TLR4-NFκB pathway

**Figure 3:**
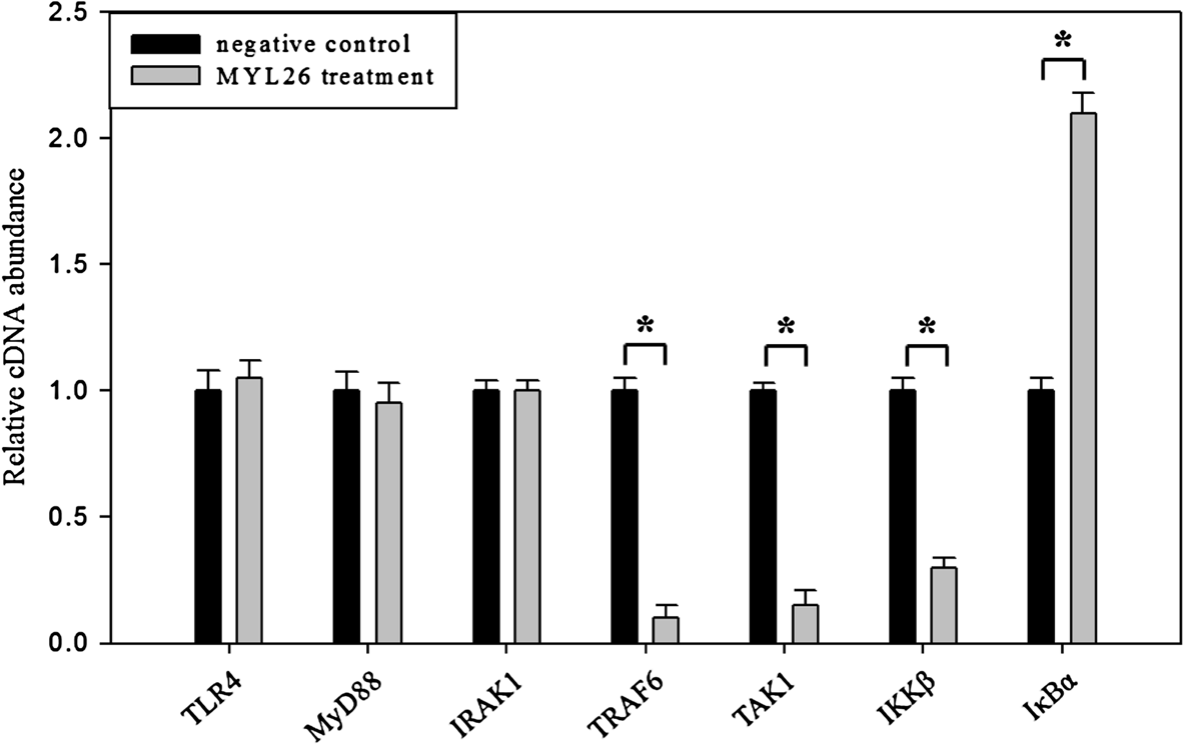
**Caco-2 cells (10**^**6 **^**cells/mL) were treated with live *L. plantarum *MYL26 (10**^**7**^ **cfu/mL) at 37°C for 10 hours followed by 1 μg/mL LPS challenge.** Gene expressions were assayed by RT-qPCT normalized by GAPDH. Symbol * represents P-value smaller than 0.05 analyzed by *t*-test in comparison with negative control group. (n = 3). Negative control: Caco-2 cells were challenged by LPS without pretreatment with probiotics.

The results of RT-qPCR (Figure 3) indicated that there are no significant differences in the expressions of *TLR4*, *MyD88* and *IRAK1* in comparison with those of LPS treatment group. The expressions of *TRAF6*, *TAK1* and *IKKβ* decreased more significantly under *L. plantarum* MYL26 treatment than those under LPS treatment alone.

### *Lactobacillus plantarum* MYL26 pretreatment elicits anti-inflammatory properties by enhancing the expressions of *TOLLIP*, *SOCS1* and *SOCS*

**Figure 4:**
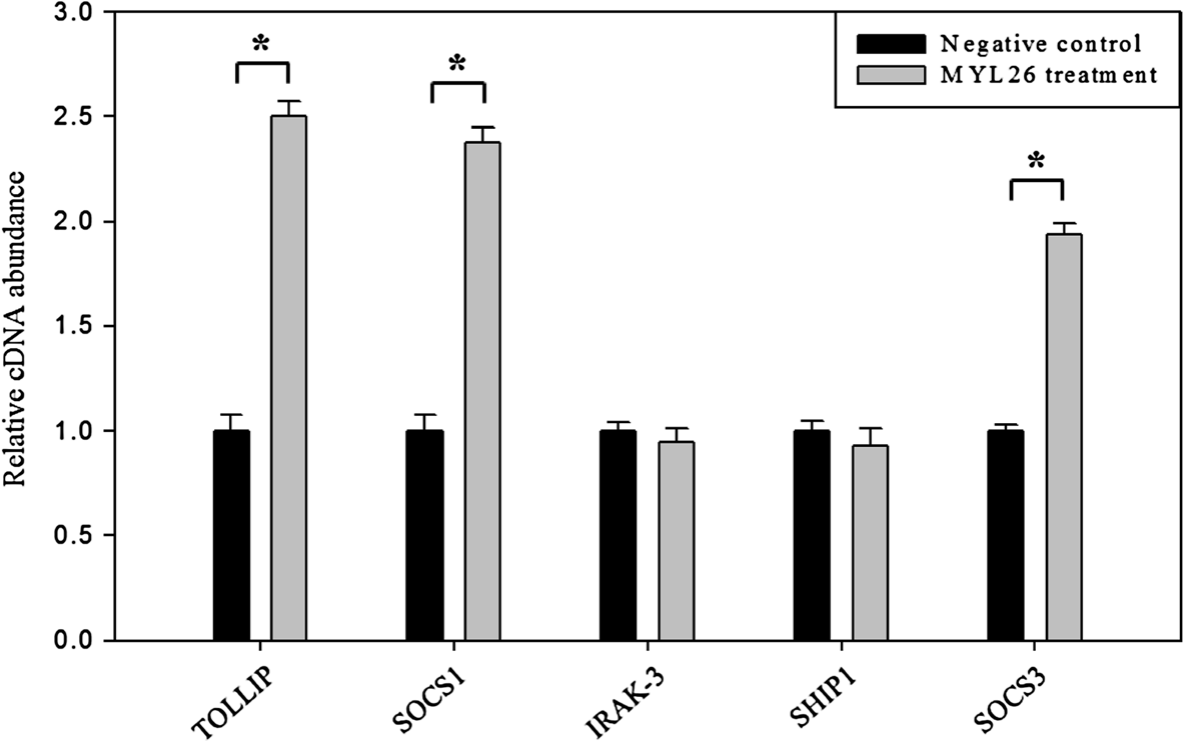
**Caco-2 cells (10**^**6 **^**cells/mL) were treated with live *L. plantarum *MYL26 (10**^**7**^ **cfu/mL) at 37°C for 10 hours.** Gene expressions were assayed by RT-qPCR normalized by GAPDH. Symbol * represents P-value smaller than 0.05 analyzed by *t*-test in comparison with negative control group. (n = 3). Negative control: Caco-2 cells were not treated with probiotics.

Since *TRAF6*, *TAK1* and *IKKβ* were down-regulated, five potential negative regulator gene expressions were examined. As shown in Figure 4, there were no considerable differences in the expressions of *IRAK3* and *SHIP1* while the expressions of *TOLLIP*, *SOCS1* and *SOCS3* were higher than those in the control groups.

### *TOLLIP*, *SOCS1* and *SOCS3* knockdown gave rise to impaired anti-inflammation abilities

**Figure 5:**
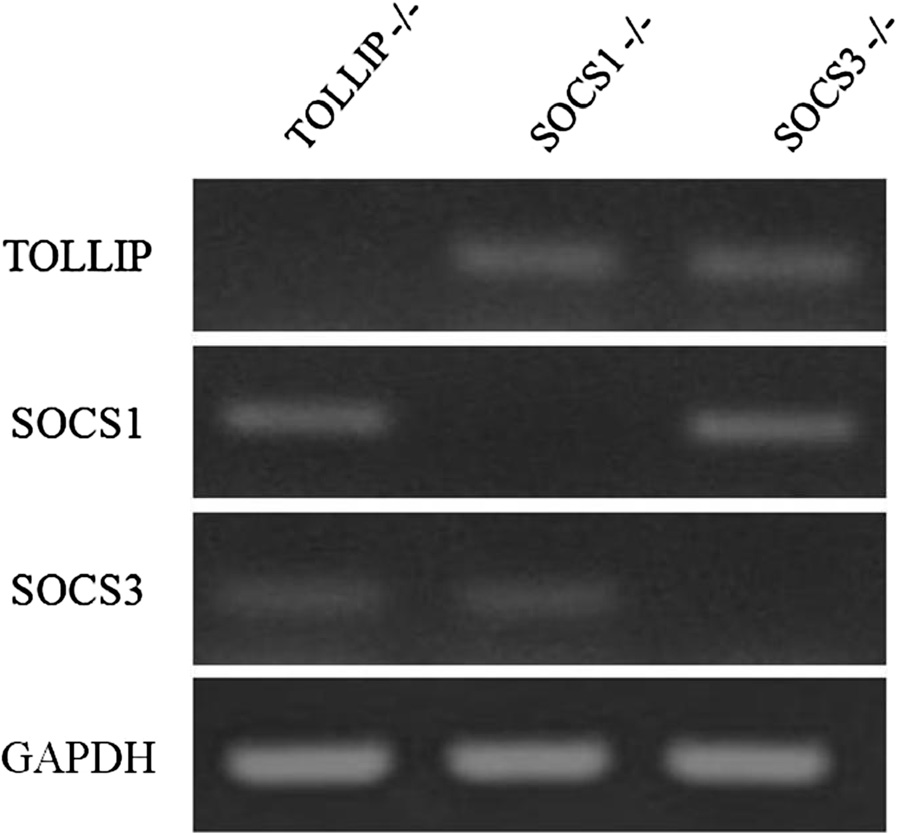
**Human *SOCS1*, *SOCS3 *and *TOLLIP *gene expressions were not off-targeted.** The siRNA experiment was conducted for 48 h.

**Figure 6:**
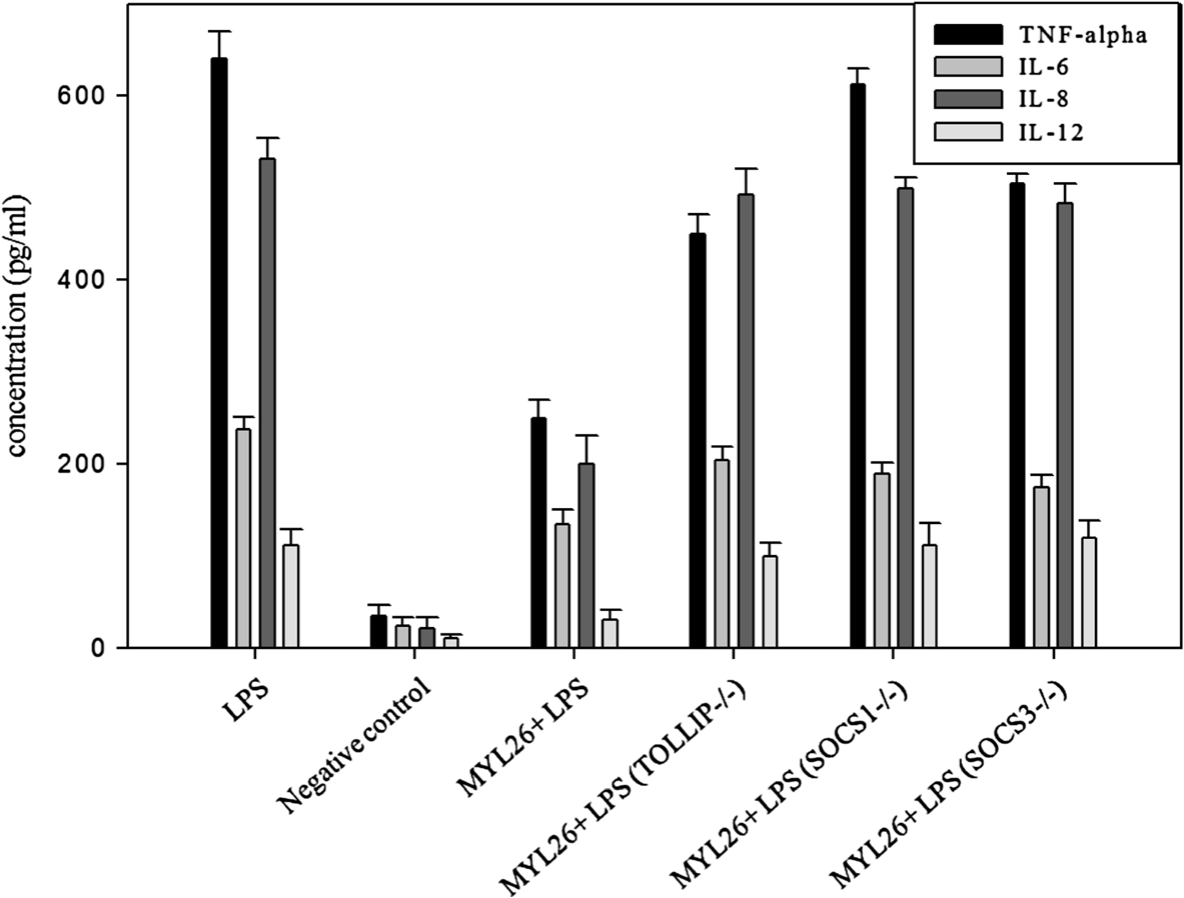
**TOLLIP, SOCS1 and SOCS3-silenced Caco-2 cells (10**^**6 **^**cells/mL) were treated with live *L. plantarum *MYL26 (10**^**7**^ **cfu/mL) at 37 ±°C for 10 hours, followed by 1 μg/mL LPS challenge.** Negative control: Caco-2 cells were not treated with LPS and probiotics. (Cytokine secretion baseline).

We then used gene knockdown technique to silence *TOLLIP*, *SOCS1* and *SOCS3*. Prior tests have shown that silencing of target genes does not decrease the expression of non-target genes (Figure 5). *TOLLIP*, *SOCS1* and *SOCS3* were silenced separately and subsequently challenged by LPS. The silencing of these three genes resulted in the partial loss of anti-inflammatory function of *L. plantarum* MYL26 (Figure 6).

### The physiologically active components that affect *SOCS1/3*, *TOLLIP* and *IκBα* expression might be located in the cell walls

**Figure 7:**
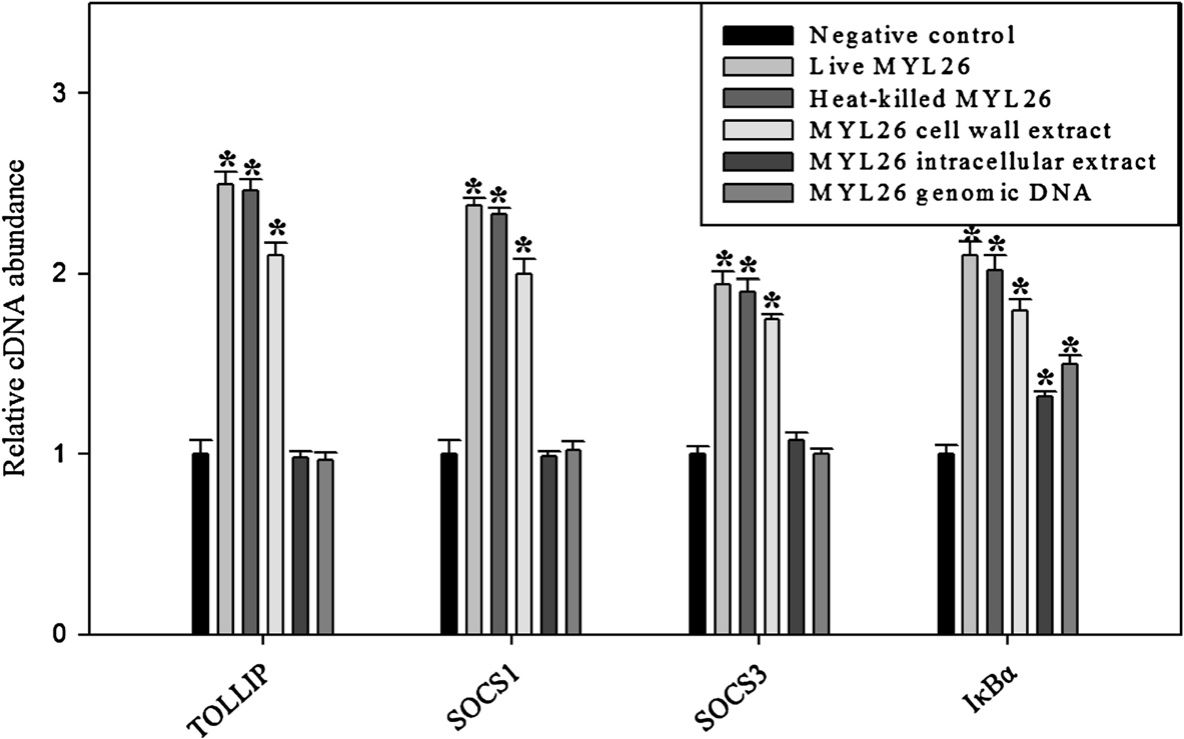
**The candidate anti-inflammation gene expressions were induced in different degrees by diverse cellular components.** Caco-2 cells (10^6^ cells/mL) were treated with live *L. plantarum* MYL26 (10^7^ cfu/mL), heat-killed bacteria (10^7^ cfu/mL), intracellular extracts (100 μg/mL), cell wall extracts (10 ± 0.2 mg/mL) and genomic DNA (1 μg/mL) at 37°C for 10 hours. Symbol * represents P-value smaller than 0.05 analyzed by *t*-test in comparison with negative control group. (n = 3). Negative control: Caco-2 cells were not treated with probiotics.

To investigate the involvement of different cellular parts in reducing LPS-induced inflammation, live bacteria, heat-killed bacteria, cell wall extract, intracellular extract and bacterial genomic DNA were tested to assess which cellular parts activate *TOLLIP*, *SOCS1*, *SOCS3* and *IκBα*. The results showed that dead *L. plantarum* MYL26 activate gene expressions as well as live bacteria. Cell wall extract, intracellular extract and genomic DNA also stimulated gene expression, but not as well as the whole cell (Figure 7).

## Discussion

Almost all of the IBD medicines are associated with decrease of inflammation signal pathways. On the other hand, pro-inflammatory cytokines play imperative character in mediating the progression of IBD. Numerous clinical trials have shown that better control of pro-inflammatory cytokine production is an essential method for improving symptoms [28-30].

Due to sustained contact with pathogen-associated molecular patterns (PAMPs), the epithelial cells act as the first barrier of defense against invading microbes. Intestinal epithelial cells take part in mediating balanced immune actions, as well as stimulating immune cells that dwell in the lamina propria. In this respect, Baumgart et al. [31] suggested that IBD results from a collapse of tolerance towards the commensal microbiota. An aberrant LPS response results in an inflammatory phenotype. As a consequence, elevated attention to probiotics for the treatment of GI tract disorders has shed light on new therapeutic regimens.

LPS tolerance may occur as the host’s defense system that confines an inflammatory break upon successive stimulation [32]. In our study, it is expected to reveal the mechanism by which prolonged contact of lactic acid bacteria with intestinal epithelial cells leads to hyporesponsive to the following inflammatory stimuli. It helps establish a probiotic screen criteria for selection of the best LPS tolerance induction bacterial strains, rather than traditional criteria focused on bile-acid resistant ability.

Until now, many possible anti-inflammatory mechanisms of probiotic actions have been proposed and it is observed that probiotic effect is both strain dependent and dose dependent [33]. Although different strains of lactic acid bacteria possess different properties, there have been the most publications reported on *L. plantarum* when searching by key words “dead probiotics” or ”killed probiotics”. As a result, we examined three different strains of *L. plantarum* and used the most potent strain MYL26, as a study object researching the underlying molecular mechanisms.

In this research, upon *L. plantarum* MYL26 treatment, the expression of genes that encode proteins participating in LPS-induced inflammation was compared with that of untreated group and found that *TRAF6*, *TAK1* and *IKKβ* expressions were suppressed. We also observed that expression of *IκBα* was increased. It was perhaps attributed to prior probiotic stimulation on Caco-2 cells, the action that caused mild inflammation (data not shown) as well as slightly NFκB nuclear translocation which encoded not only cytokines but also IκBα. This observation was similar to the results Wahlstrom et al. reported [34]. They suggested that low-dose LPS pretreatment changed subsequent LPS-activated signal transduction pathways by means of up-regulation of *IκBα* that acted as a feedback control inhibitor.

Since the results showed that anti-inflammatory effects of *L. plantarum* MYL26 on Caco-2 might be through interfering with TLR4 downstream pathway, it is reasonable to infer that the activation of the negative regulators of TLR4-NFκb pathway contributes to the anti-inflammatory effect. We investigated TLRs-associated negative regulators, including *TOLLIP*, *SOCS1*, *SOCS3, IRAK3* and *SHIP1,* and found *TOLLIP* and *SOCS1/3* expressions were enhanced by *L. plantarum* MYL26 treatment. However, the consequence that *TOLLIP* and *SOCS1/3* knockdown gave rise to impaired anti-inflammatory ability further supported the hypothesis that activation of the negative regulators of TLR4-NFκb pathway is a primary exploit for the anti-inflammatory effect *L. plantarum* MYL26 exerts. As numerous literatures have revealed that different components of the bacterial cells can result in different activities on the human GI tract, such as cell wall components [35], genomic DNA [36], and intracellular extract [37], we further researched the *TOLLIP* and *SOCS1/3* activation ability of live/ heat-killed whole bacterial cells, cell wall extract, intracellular extract and genomic DNA from *L. plantarum* MYL26 to see which cellular parts contributed mostly to LPS tolerance induction.

In contrast with our expectations, although intracellular extract and genomic DNA induced *IκBα* expression more significantly than that of control group, they failed to activate *TOLLIP*, *SOCS1*, and *SOCS3*. There are five TLRs (TLR2/ 4/ 5/ 7/ 9) sharing similar downstream signal pathway (MyD88, IRAK, TRAF, IKK, NFκb) [38]. Except for *IκBα* which directly binds to NFκb, the negative regulators *TOLLIP*, *SOCS1*, and *SOCS3* are well-established having abilities in interference with recruitment of MyD88 and IRAK. It has been reported that *TOLLIP*, *SOCS1*, and *SOCS3* not only attenuate TLR4 signaling, but also have impact on TLR2/5/7/9 signaling [39,40]. Briefly, *L. plantarum* MYL26 intracellular extract and genomic DNA activate TLRs-NFκb pathways other than TLR4 (TLRs cross-tolerance), but they did not attenuate inflammation through induction of *TOLLIP*, *SOCS1*, and *SOCS3.* Taken together, we proposed that *L. plantarum* MYL26 intracellular extract and genomic DNA induced LPS tolerance through pathways different from induction of *Tollip*, *SOCS-1* and *SOCS-3*, which were key negative regulators activated by live/dead *L. plantarum* MYL26 and cell wall components.

One of the limitations of this study is that the causes of IBD, other than breakdown of LPS tolerance, are multifaceted. Several lines of evidence has pointed out that in addition to inherited factors, pollution, drugs, diets, breastfeeding, even emotional stress, could be responsible for genetically failing to interpret molecular microbial patterns appropriately, thus leading to irregular innate and adaptive immune responses [41,42]. The second limitation is that PAMPs other than LPS induce GI inflammation through different pathways. Criteria for probiotic selection of LPS tolerance induction strains might be not suitable with respect to inflammation symptoms caused by other PAMPs.

## Conclusions

The administration of lactic acid bacteria in patients suffering from GI disorders regularly depends on try-error methods, and numerous probiotics treatment applied to clinical trials showed frustrated results, which perhaps might be related to the fact that the probiotic screening criteria is generally based on susceptibility to artificial GI environments (acid and bile resistance) or adhesive properties instead of on immunomodulatory capacities, for instance, induction of LPS tolerance. Our research provided a new insight to describe the *L. plantarum* strain-dependent characterization in terms of anti-inflammatory effects, and suggested an essential role for *Lactobacillus plantarum* and *Lactobacillus plantarum*-derived constituents in the induction of LPS tolerance.

## Competing interests

The authors declare that they have no competing interest.

## Authors’ contributions

Chiu YH and Lin MY conceived and designed the experiments. Tsai CC and Huang CT performed the experiments. Lu YC, Ou CC and Lin SL analyzed the data and performed the computational analysis, producing the figures and tables. Chiu YH drafted the manuscript and Lin MY revised it. All authors read and approved the final manuscript.
